# Scheduled Bronchoscopy with Nebulized Heparin and N-Acetylcysteine in Burn Patients with Inhalation Injury: A Randomized Trial

**DOI:** 10.3390/ebj7020022

**Published:** 2026-04-29

**Authors:** Thai Ngoc Minh Nguyen, Nhu Lam Nguyen, Dinh Hung Tran

**Affiliations:** Viet Nam National Burn Hospital, Hanoi 100000, Vietnam; minhnguyennib@gmail.com (T.N.M.N.);

**Keywords:** inhalation injury, bronchoscopy, nebulized heparin, N-acetylcysteine, driving pressure, static compliance

## Abstract

**Highlights:**

**What are the main findings?**

- The combination of scheduled therapeutic bronchoscopy with nebulized heparin and N-acetylcysteine significantly reduced driving pressure and doubled static respiratory compliance by day 7.
- Unadjusted 28-day mortality was lower in the intervention group, although baseline injury severity remained the dominant independent predictor of survival in multivariable analysis.

**What are the implications of the main findings?**

- Proactive mechanical and pharmacological airway clearance safely mitigates ventilator-induced lung injury by restoring functional lung volume in severe inhalation injury.
- This multimodal strategy serves as a physiologically beneficial adjunct in managing acute respiratory failure from inhalation injury without causing systemic anticoagulation.

**Abstract:**

Inhalation injury (II) exacerbates burn mortality via obstructive fibrin casts. We evaluated a protocol combining scheduled flexible bronchoscopy (FOB) with nebulized heparin and N-acetylcysteine (NAC). This single-center, randomized controlled trial enrolled 76 mechanically ventilated adult burn patients with bronchoscopically confirmed II. The intervention (*n* = 38) comprised a 7-day protocol of scheduled FOB with alternating nebulized heparin (5000 IU) and 20% NAC every 4 h. Controls (*n* = 38) received standard care with on-demand FOB. Primary outcomes were 28-day mortality and day-7 Lung Injury Score (LIS). Unadjusted 28-day mortality was lower in the intervention group (57.9% vs. 81.6%; *p* = 0.025), alongside a decreased median day-7 LIS (1.0 vs. 1.38; *p* = 0.021). Respiratory mechanics improved significantly, demonstrating reduced driving pressure and increased static compliance (*p* < 0.001). However, in multivariable Cox regression, baseline injury severity independently predicted mortality, while the intervention indicated a non-significant hazard reduction trend (aHR = 0.66, 95% CI: 0.36–1.23). No systemic anticoagulation occurred. In conclusion, scheduled FOB with nebulized heparin and NAC improves respiratory mechanics and attenuates lung injury in II. Although unadjusted mortality decreased, baseline severity remains the primary mortality driver, suggesting this protocol is a physiologically beneficial adjunct requiring further multicenter validation. Trial registration: Thai Clinical Trials Registry, TCTR20260408001 (retrospectively registered).

## 1. Introduction

The combination of major cutaneous burns and inhalation injury (II) presents a critical clinical challenge, independently increasing expected mortality by up to 20% above that predicted by burn size and age alone [1,2]. The pathophysiology of II is characterized by thermal damage, chemical lower airway inflammation and systemic toxicity. This cascade culminates in the denudation of the respiratory epithelium and the formation of obstructive tracheobronchial fibrin casts [3,4,5]. These casts promote atelectasis, increase the risk of barotrauma, and serve as a nidus for ventilator-associated pneumonia (VAP), thereby driving a vicious pathophysiological cycle of pulmonary deterioration [6,7].

Flexible fiberoptic bronchoscopy (FOB) remains the cornerstone for the mechanical clearance of these obstructive casts [8]. However, its therapeutic benefits are often transient; rapid cast re-accumulation is driven by persistent local inflammation and the activation of the coagulation cascade [9]. Pharmacological strategies utilizing nebulized unfractionated heparin target this procoagulant environment by potentiating antithrombin to inhibit fibrin formation, while N-acetylcysteine (NAC) acts synergistically as a mucolytic and antioxidant agent [10,11,12]. While pediatric studies have demonstrated survival benefits when combining these agents with frequent pulmonary toilet [13], data in adult cohorts remain conflicting. This discrepancy may largely stem from inadequate distal drug delivery through already obstructed airways [14,15].

We hypothesized that a proactive, synergistic protocol—integrating scheduled mechanical clearance via therapeutic FOB with localized pharmacological modulation (nebulized heparin and NAC)—would facilitate distal drug delivery, prevent cast reformation, and consequently improve both clinical and physiological outcomes. Therefore, this randomized controlled trial aimed to evaluate the efficacy of this combined protocol on 28-day mortality and the severity of lung injury. Furthermore, we conducted a detailed mechanistic analysis to assess its impact on key respiratory mechanics, specifically driving pressure (ΔP) and static respiratory system compliance (Crs), which are central determinants of ventilator-induced lung injury and patient survival [16].

## 2. Materials and Methods

### 2.1. Study Design and Ethics

A single-center, prospective, randomized, open-label, controlled superiority trial was conducted at the Vietnam National Burn Hospital (Hanoi, Vietnam) between November 2021 and April 2024. The study protocol was approved by the hospital’s Institutional Review Board (protocol code 250/IRB; approved on 26 May 2021), and the trial was retrospectively registered on the Thai Clinical Trials Registry (TCTR20260408001). Written informed consent was obtained from all patients’ legal surrogates prior to enrollment. The CONSORT 2025 checklist for this trial is provided as Supplementary Material (File S1).

### 2.2. Participants

Patients were eligible for inclusion if they were aged 18 to 65 years, had sustained acute thermal burns, and presented with bronchoscopically confirmed II within 24 h of admission requiring invasive mechanical ventilation. In the context of massive thermal burns, the presence of any bronchoscopically confirmed II enough to precipitate acute respiratory failure and necessitate endotracheal intubation inherently constitutes a critical pulmonary insult. Nevertheless, to provide a comprehensive descriptive profile of baseline airway damage, the severity of II for all eligible patients was systematically categorized according to the Abbreviated Injury Score for inhalation (Bronchoscopic Grades 1 to 4) [5,17].

Criteria for exclusion comprised the presence of a bleeding diathesis or concurrent therapeutic anticoagulation, severe chronic lung disease (GOLD 3–4 COPD), a known allergy to heparin or NAC, pregnancy, or an expected survival of less than 72 h as determined by a consensus of two senior intensivists.

Burn depth was initially assessed by experienced burn surgeons upon admission and sequentially re-evaluated throughout the first 48 h of resuscitation. For the purposes of this study, the term ‘deep burn’ was defined as deep partial-thickness and full-thickness burns that necessitated early surgical excision.

### 2.3. Randomization and Interventions

Participants were randomized in a 1:1 ratio using sealed, opaque envelopes. Due to the nature of the intervention, the trial was open-label, and blinding of the treating clinical team was not feasible. However, to minimize detection bias, outcome adjudicators for the primary endpoints (LIS and VAP) and the trial statistician were strictly blinded to group allocation. Secondary outcomes were extracted from electronic medical records using objective criteria.

Intervention group (*n* = 38): Patients received an initial therapeutic FOB followed by scheduled therapeutic FOBs (performed daily for the first 3 days, then on alternate days). Concurrently, they received nebulized unfractionated heparin (5000 IU in 3 mL normal saline) and NAC (3 mL of 20% solution), administered in an alternating schedule every 4 h for 7 days via a vibrating mesh nebulizer (Aerogen ProX, Aerogen Ltd., Galway, Ireland) integrated into the ventilator circuit.

Control group (*n* = 38): Patients received an initial diagnostic FOB followed by standard airway management, which included routine airway humidification, chest physiotherapy, and on-demand endotracheal suctioning. Subsequent FOBs were performed only as clinically indicated (e.g., for lobar atelectasis). No scheduled FOB or protocolized nebulization was performed.

Standard of care and co-interventions (both groups): All patients received identical protocols for burn shock resuscitation (Parkland formula), VAP prophylaxis, analgesia, sedation, and nutrition in accordance with international guidelines [7,18,19]. Mechanical ventilation was managed using a lung-protective strategy: tidal volume was set at 6–8 mL/kg of predicted body weight, and plateau pressure (Pplat) was maintained at <30 cmH_2_O. Positive end-expiratory pressure (PEEP) was individually titrated to maintain SpO_2_ ≥ 92% while concurrently minimizing Pplat. The administration of short-acting bronchodilators (e.g., albuterol or ipratropium) was standardized across both cohorts, guided strictly by the clinical presence of bronchospasm. Daily fluid infusion rates were systematically recorded for all patients to account for resuscitation volumes.

The bronchoscopy protocol was standardized and performed by attending intensivists to ensure procedural consistency. Sequential bronchoalveolar lavage was conducted using 10–20 mL aliquots of warm (37 °C) normal saline, with a total volume not exceeding 100 mL per session, to facilitate the removal of soot and obstructive fibrin casts. Adequate sedation and analgesia were maintained during the procedure using continuous infusions of midazolam combined with fentanyl.

Standardized ICU co-interventions were applied equally to both groups according to institutional protocols. This included continuous sedation, closed-system endotracheal suctioning, and routine chest physiotherapy. Other aspects of critical care, including fluid resuscitation and targeted temperature management, followed the standard of care for severe burns.

No patient received systemic pharmacological VTE prophylaxis (e.g., low-molecular-weight heparin), per institutional protocol for massive burns due to the high bleeding risk from raw wounds and early surgical excisions.

### 2.4. Study Endpoints

The primary endpoints of this trial comprised 28-day all-cause mortality and the severity of pulmonary impairment, as quantified by the Lung Injury Score (LIS) at day 7. The LIS was calculated using the Murray score based on four variables: chest radiograph consolidation, PaO_2_/FiO_2_ ratio, positive end-expiratory pressure (PEEP), and respiratory system compliance [20].

Secondary clinical outcomes evaluated the incidence of ventilator-associated pneumonia (adjudicated using the CDC/NHSN surveillance definitions), the number of ventilator-free days at day 28, total duration of mechanical ventilation, and intensive care unit length of stay. Additionally, we monitored the incidence of subsequent severe complications, including septic shock, acute kidney injury (KDIGO stage ≥ 2), and acute respiratory distress syndrome (based on the Berlin definition) [21,22,23,24].

To address the mechanistic objectives, key respiratory system parameters, specifically driving pressure (ΔP = Pplat − PEEP) and static compliance (Crs = Vt/[Pplat − PEEP]), were documented at baseline and subsequently reassessed on day 7. Finally, safety endpoints encompassed systemic coagulation profiles (PT, aPTT, INR, and fibrinogen) at day 7, along with the strict prospective tracking of any procedure-related adverse events, such as significant airway hemorrhage, pneumothorax, or severe peri-procedural hypoxemia.

### 2.5. Statistical Analysis

The sample size was calculated to detect a 30% absolute reduction in 28-day mortality, decreasing from an expected 80% in the control group to 50% in the intervention group. The baseline 80% mortality estimate was derived from institutional historical data for patients with severe inhalation injury and corroborated by established predictive models [1,25]. To achieve 80% power with a two-sided α of 0.05, a total of 76 patients (38 per group) was required.

Continuous variables were reported as mean (standard deviation, SD) or median (interquartile range, IQR), depending on their distribution, assessed by the Shapiro–Wilk test. Categorical variables were presented as frequencies and percentages. Between-group comparisons were performed using the Chi-square test or Fisher’s exact test for categorical variables, and Student’s *t*-test or Mann–Whitney U test for continuous variables (including total daily fluid volumes), as appropriate. Within-group changes from baseline to day 7 were evaluated using the Wilcoxon signed-rank test.

Changes in respiratory mechanics (ΔP, Crs) and LIS over time were analyzed using linear mixed-effects models with an unstructured covariance structure. Each model included group, time (as a continuous variable), and the group-by-time interaction as fixed effects, alongside a random intercept for each patient to account for within-subject correlation. The interaction term was used to test whether the trajectory of change differed between the two groups. Results are presented as estimated marginal means with 95% confidence intervals.

Survival time was defined as the number of days from admission to death from any cause within the first 28 days; patients alive at day 28 were censored. The Kaplan–Meier method was used to estimate 28-day survival probabilities, and the log-rank test was employed for unadjusted between-group comparisons.

To identify predictors of 28-day mortality while strictly avoiding model overfitting (given 53 events) and immortal time bias, we constructed a parsimonious multivariable Cox proportional hazards model. Time-dependent variables, most notably the duration of mechanical ventilation, were intentionally excluded. The model was streamlined to include only the most fundamental baseline clinical covariates: intervention group, age, sex, total body surface area (TBSA), deep burn area, and bronchoscopic inhalation injury grade. Adjusted hazard ratios (aHRs) with 95% CIs were calculated. All statistical tests were two-sided, and a *p*-value < 0.05 was considered statistically significant [26]. Analyses were conducted using Stata version 18.0 (StataCorp, College Station, TX, USA).

## 3. Results

### 3.1. Participant Flow and Baseline Characteristics

**Figure 1:**
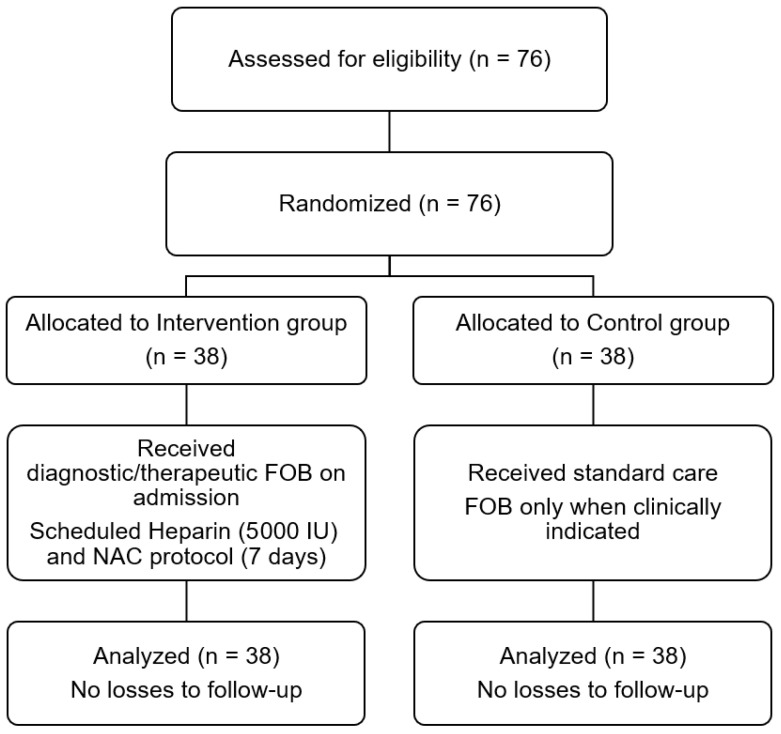
CONSORT Flow Diagram of participant enrollment, randomization, and analysis.

All 76 randomized patients completed the study (Figure 1, CONSORT Flow Diagram). Baseline characteristics were well balanced between the two groups (Table 1). The cohort exhibited extreme injury severity, characterized by a median TBSA of >60% and a deep burn area of approximately 40%, commonly presenting with metabolic acidosis and severe hypoxemia at admission. To ensure that fluid management did not confound pulmonary outcomes, daily fluid resuscitation volumes were systematically tracked. There were no significant between-group differences in the average infusion rates during the first 24 h or in the total daily fluid volumes administered from Day 2 through Day 7 (Table 2).

### 3.2. Primary Clinical Outcomes

**Figure 2:**
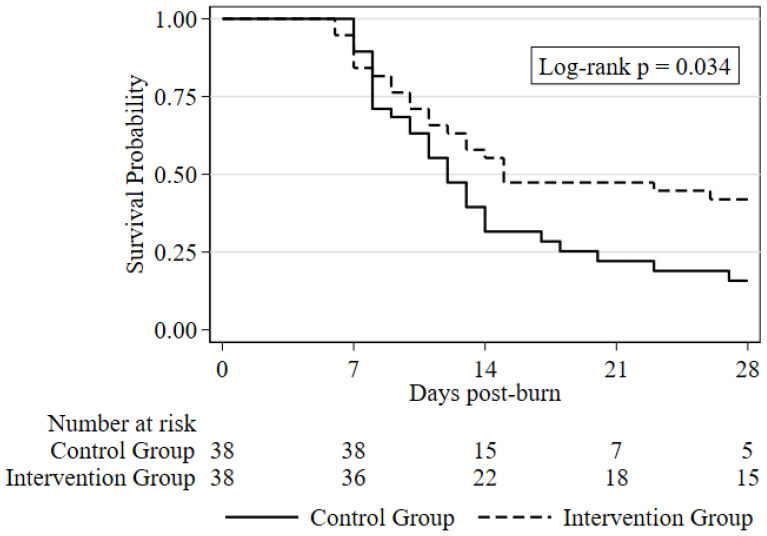
Kaplan–Meier curves for 28-day survival in the intervention and control groups.

The 28-day all-cause mortality rate was significantly lower in the intervention group compared to the control group (57.9% [22/38] vs. 81.6% [31/38], respectively; *p* = 0.025). This corresponds to an absolute risk reduction of 23.7% (95% CI: 3.7% to 43.6%). Furthermore, Kaplan–Meier survival analysis demonstrated a significant divergence in survival trajectories favoring the intervention group (log-rank *p* = 0.034) (Figure 2).

**Figure 3:**
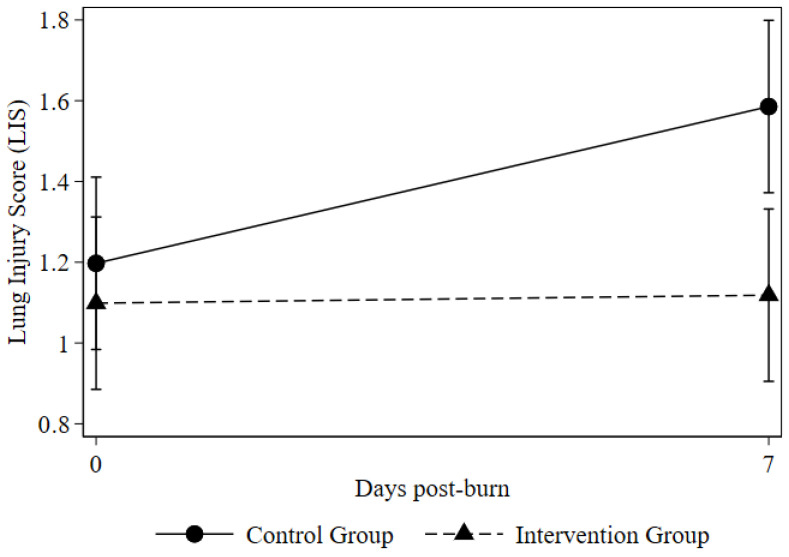
Lung Injury Score at baseline and day 7 in the intervention and control groups.

Regarding the severity of pulmonary impairment, both cohorts exhibited comparable Lung Injury Scores (LIS) at baseline. However, by Day 7, patients in the intervention group achieved a significantly lower median LIS (1.0, IQR 0.5–1.75) relative to the control group (1.375, IQR 1.0–2.25; *p* = 0.021). Consistent with this unadjusted comparison, the linear mixed-effects model revealed a strong trend toward a more substantial reduction in LIS over time in the intervention group (group-by-time interaction *p* = 0.091). The estimated marginal means for LIS trajectories are illustrated in Figure 3 (estimates provided in Table 3).

### 3.3. Mechanistic Outcomes: Respiratory Mechanics

**Figure 4:**
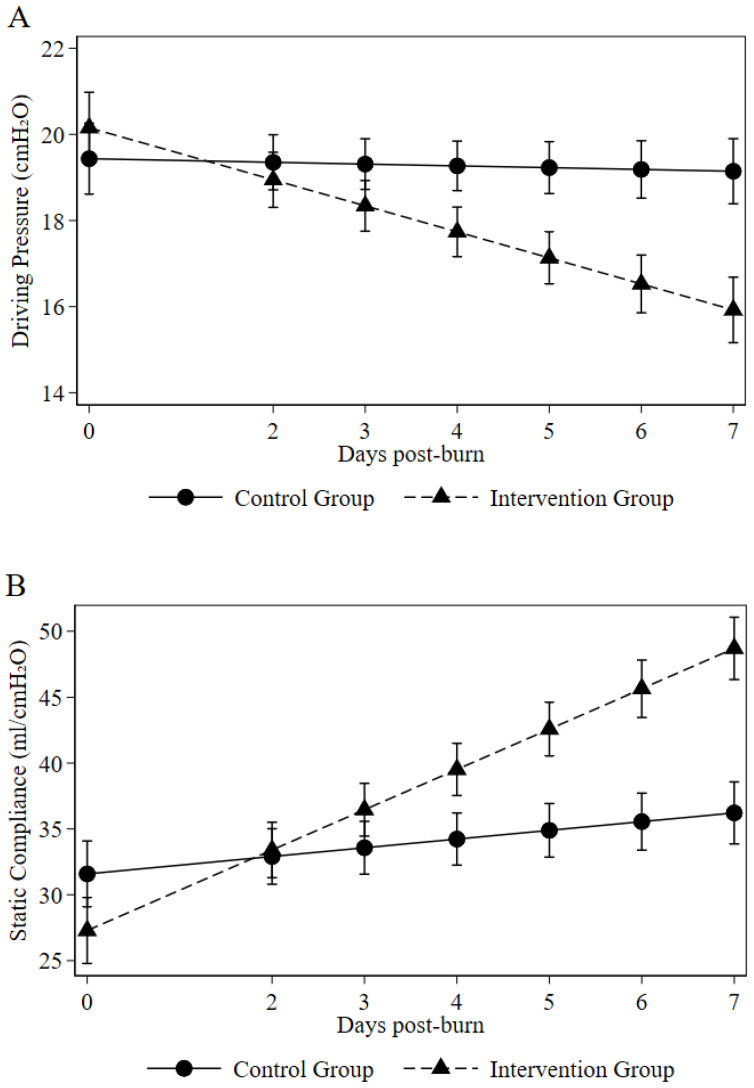
Changes in respiratory mechanics over time. Predicted trajectories (with 95% confidence bands) from linear mixed-effects models for (**A**) driving pressure and (**B**) static compliance in the control (solid line) and intervention (dashed line) groups. The group-by-time interaction was significant for both parameters (*p* < 0.001), indicating greater improvement in the intervention group.

To assess the physiological impact of the protocol, we analyzed the trajectories of driving pressure (ΔP) and static compliance (Crs) using linear mixed-effects models. At baseline, there were no significant differences in respiratory mechanics between the two groups (Table 3). Over the 7-day study period, the intervention group demonstrated a significantly greater physiological improvement in both parameters compared to the control group, evidenced by highly significant group-by-time interactions (both *p* < 0.001; Table 3 and Figure 4). Specifically, the estimated mean ΔP decreased from 19.4 cmH_2_O at day 0 to 15.9 cmH_2_O at day 7 in the intervention group, whereas it remained elevated at approximately 19.4 cmH_2_O in the control group. Concurrent with this, Crs increased substantially from 31.6 to 48.7 mL/cmH_2_O in the intervention group, with only a modest change observed in the control group (from 31.6 to 36.2 mL/cmH_2_O). These findings indicate that the proactive airway management protocol facilitates a substantial and sustained improvement in respiratory mechanics. Alongside the improvement in respiratory mechanics, systemic oxygenation demonstrated a remarkably favorable trajectory in the intervention group. While baseline PaO_2_/FiO_2_ ratios were comparable between the control and intervention groups (144 [IQR 81–216] vs. 173 [IQR 112–242] mmHg; *p* = 0.125), patients receiving the proactive airway protocol exhibited a significantly higher PaO_2_/FiO_2_ ratio by Day 7 compared to standard care (286 [IQR 210–350] vs. 156 [IQR 112–210] mmHg; *p* < 0.001). This indicates a profound restoration of gas exchange accompanying the resolution of airway obstruction.

### 3.4. Secondary Clinical and Safety Outcomes

Clinical outcomes are summarized in Table 4. A consistent trend toward reduced secondary complication rates was observed in the intervention group, including ventilator-associated pneumonia (50.0% vs. 68.4%; *p* = 0.102), ARDS (31.6% vs. 47.4%; *p* = 0.151), and acute kidney injury (23.7% vs. 36.8%; *p* = 0.201), although these differences did not reach statistical significance. The incidence of septic shock was comparable between the two cohorts (60.5% vs. 65.8%; *p* = 0.634). Ventilator-free days at day 28 were also comparable (median 0 in both groups; *p* = 0.145), largely reflecting the survivorship bias inherent to a cohort with extreme overall injury severity and high mortality. Safety analysis revealed no significant differences in systemic coagulation parameters between the groups at day 7 (Table 5), confirming the localized effect of nebulized heparin. Furthermore, procedure-related complication rates during bronchoscopy were low and comparable between the cohorts.

### 3.5. Multivariate Analysis

To rigorously identify predictors of 28-day mortality while avoiding immortal time bias and model overfitting (as flagged in prior models that included time-dependent variables such as ventilation duration), we evaluated a parsimonious multivariable Cox proportional hazards model using six fundamental baseline covariates.

In this rigorously adjusted analysis, established markers of injury acuity emerged as significant independent predictors of mortality: older age (adjusted HR = 1.04 per year; 95% CI: 1.01–1.07; *p* = 0.005), larger TBSA (adjusted HR = 1.03 per %; 95% CI: 1.00–1.05; *p* = 0.030), and a higher grade of inhalation injury (adjusted HR = 1.61 per grade; 95% CI: 1.03–2.53; *p* = 0.037).

While the unadjusted 28-day mortality rate was significantly lower in the intervention group (Table 4), the intervention did not reach statistical significance as an independent predictor in the multivariable model (adjusted HR = 0.66; 95% CI: 0.36–1.23; *p* = 0.189). This indicates that, in this cohort characterized by extreme acuity, overall survival is predominantly determined by baseline physiological reserve and the magnitude of the initial burn insult, although the proactive airway protocol maintained a clinically meaningful trend toward mortality hazard reduction (a 34% relative reduction). Full results are detailed in Table 6.

## 4. Discussion

The present randomized controlled trial suggests that, a proactive, multimodal airway management strategy, which integrates scheduled therapeutic bronchoscopy with the localized delivery of nebulized heparin and N-acetylcysteine (NAC), may represent a beneficial approach in the treatment of II. The observed 23.7% absolute reduction in unadjusted 28-day mortality occurred in a cohort characterized by severe baseline injuries, where predicted mortality typically exceeds 80% [1,25,27]. This initial finding suggests that the protocol may help mitigate the pathophysiological progression of II before irreversible parenchymal failure occurs. However, it is crucial to contextualize this unadjusted survival benefit within the multivariable framework. The median admission carboxyhemoglobin (CO-Hb) level was notably low (1.5%, IQR 0–11%) despite the high prevalence of severe inhalation injury. As previously reported, this discrepancy primarily stems from prolonged pre-hospital transport times and inconsistent early respiratory support [28]. Consequently, admission CO-Hb values likely reflect survivorship bias. Furthermore, our parsimonious Cox regression analysis revealed that survival in this extreme cohort is predominantly driven by baseline injury severity—specifically age, TBSA, and the initial grade of inhalation injury. In this adjusted model, the intervention did not reach statistical significance as an independent predictor of mortality (aHR = 0.66; 95% CI: 0.36 to 1.23; *p* = 0.189). Therefore, the unadjusted survival benefit must be interpreted with caution. Crucially, our mechanistic findings identify the physiological substrate of this survival advantage, demonstrating that the benefit is fundamentally mediated by a robust restoration of respiratory mechanics. By mitigating the mechanical burden of fibrin casts, the protocol appears to protect functional lung units from high driving pressures [16,29].

The pathophysiology of II is characterized by a self-perpetuating cycle of airway obstruction, subsequent atelectasis, and regional lung overdistension [3,4,5]. Fibrin casts function as pathological check valves that permit inspiratory inflow but impede expiratory egress, which induces localized air trapping and excessive alveolar strain even in cases where global airway pressures remain within clinically acceptable limits [16,29,30]. The protocol combines mechanical clearance via FOB with pharmacological modulation. Scheduled FOB provides critical mechanical clearance of proximal obstructions, serving the dual purpose of relieving immediate airway blockage and restoring conduit patency necessary for the effective distal delivery of nebulized heparin and NAC [8,9,31]. This optimized delivery ensures that the pharmacological agents reach the pulmonary microenvironment at the precise locations where fibrin polymerization and oxidative stress are most pronounced [10,11,12,31,32]. Subsequent scheduled FOB sessions then facilitate the evacuation of pharmacologically liquefied secretions, thereby preventing the reformation and organization of obstructive casts. This combined approach may address the limitations of nebulization alone, as seen in previous studies where existing obstructions likely prevented distal drug delivery [15]. In the HIHI trial, the absence of protocolized mechanical clearance may have significantly compromised the regional bioavailability of nebulized heparin within the most severely affected lung segments, where pre-existing cast formation would have prevented any therapeutic penetration of aerosolized droplets.

The physiological impact of our protocol is most clearly reflected in the marked improvement in driving pressure (ΔP) and static compliance (Crs). The median ΔP decreased from 19.4 to 15.9 cmH_2_O in the intervention group, which represents a pivotal finding because a threshold of 15 cmH_2_O is widely recognized in the ARDS literature as a critical transition point for survival [16,33,34]. Driving pressure serves as a functional surrogate for the cyclic lung strain imposed on aerated lung tissue, and its reduction suggests a significant decrease in the mechanical energy delivered to the pulmonary parenchyma [16,29,30]. By substantially lowering ΔP, our protocol likely mitigates the risk of VILI and protects the functional ‘baby lung’ from the deleterious effects of repetitive overdistension [29]. The significant increase in static compliance, from 31.6 to 48.7 mL/cmH_2_O, is particularly striking compared to the modest changes observed in the control group. In II, the loss of compliance is traditionally viewed as a progressive or even irreversible process, driven by extensive cast-related atelectasis, secondary surfactant dysfunction, and the development of high-permeability pulmonary edema [5,34,35,36]. Our data suggest that a substantial portion of this compliance loss is, in fact, reversible through the synergy of aggressive mechanical clearance and local anticoagulation. Our data also suggest that the significant improvement in static compliance is likely due to the sustained recruitment of previously obstructed alveoli [37,38].

The significant survival advantage observed in the intervention group occurred despite statistically comparable incidences of subsequent VAP, septic shock, and ARDS. This apparent paradox is resolved when examining the physiological data. By aggressively clearing obstructive casts, the protocol preserved respiratory mechanics, mitigated ventilator-induced lung injury (VILI), and profoundly improved overall oxygenation, as evidenced by the near-doubling of the PaO_2_/FiO_2_ ratio by Day 7 (median 286 vs. 156 mmHg). This enhanced physiological reserve may have provided a better physiological buffer, potentially allowing patients to tolerate secondary clinical complications that proved fatal in the control group.

One potential confounder in II trials is variability in fluid resuscitation, which can exacerbate pulmonary edema and cloud the interpretation of respiratory mechanics [4,18]. In severe inhalation injury, fluid demands are inherently elevated due to systemic capillary leak, requiring resuscitation to be titrated to clinical endpoints rather than relying solely on admission weight. To minimize confounding effects on respiratory data, our lung-protective ventilation strategy was standardized using predicted body weight for tidal volume calculations (6 to 8 mL/kg). This methodological standardization ensures that the significant physiological improvements observed in driving pressure and static compliance are robustly attributable to therapeutic airway clearance and local pharmacological modulation, rather than variations in patient anatomy. Furthermore, the high mortality in the control group (81.6%) reflects the natural history of severe inhalation injury combined with thermal trauma [1,25,39]. The lack of a significant difference in ventilator-free days and the observed trend toward longer ventilation in survivors (15 vs. 12 days, *p* = 0.098) must be interpreted through the lens of survivorship bias [40,41,42,43]. In a cohort with such high predicted mortality, a life-saving intervention creates a group of survivors who are among the most severely injured. These patients face prolonged ventilation due to the sequelae of massive burns, such as repeated surgeries and sepsis, rather than ongoing pulmonary failure. Thus, outcomes conditional on survival, such as ventilator-free days, can be misleading in high-mortality populations [41,42,43]. This is further supported by our multivariate Cox model, where the paradoxical finding of total ventilator days being a protective factor (HR 0.85, *p* < 0.001) reflects that only those surviving the acute phase could accumulate a longer duration of mechanical ventilation.

Safety remains a paramount concern when administering high-dose anticoagulants. Despite a daily dose of 30,000 IU of nebulized heparin, we found no evidence of systemic anticoagulation, confirming that the drug remains compartmentalized within the pulmonary system [44,45]. This safety profile, combined with the profound survival benefit, suggests that while resource-intensive, this multimodal protocol warrants consideration as a viable adjunctive strategy in high-volume burn centers. Future research should now focus on determining the cost-effectiveness and feasibility of implementing this clear–treat–clear paradigm in varied clinical settings [46,47].

Our study has several important limitations. First, the single-center, open-label design may introduce performance and detection biases, although we mitigated these risks by utilizing blinded adjudicators for key endpoints. Second, because our protocol is a multimodal intervention, it is impossible to isolate the individual therapeutic contributions of scheduled FOB, nebulized heparin, or NAC. Third, our mechanistic analysis evaluated parameters at only two time points (Day 0 and Day 7), which potentially introduced survivorship bias as Day 7 data were restricted to survivors. This limited temporal resolution also reduced statistical power, likely explaining the slight discrepancy in LIS improvement between the mixed-model interaction and the unadjusted Day 7 comparison. Fourth, we did not systematically collect data on specific causes of death, limiting our ability to determine if the intervention preferentially reduced mortality from respiratory failure versus sepsis. Finally, due to administrative constraints during the COVID-19 pandemic at the time of study initiation, this trial was retrospectively registered on the Thai Clinical Trials Registry (TCTR20260408001). Nevertheless, the protocol was executed in full compliance with the original ethical approval granted by the Institutional Review Board of the Vietnam National Burn Hospital.

Based on these findings and limitations, we propose a clear research agenda to validate and refine this approach. First, a definitive, prospectively registered, multicenter, double-blind, placebo-controlled RCT is needed [46]. To address the multimodal nature of our protocol, future studies should consider a factorial design to isolate individual therapeutic contributions [47]. Crucially, future designs must incorporate continuous serial measurements of key respiratory mechanics with greater temporal resolution (e.g., daily) to fully characterize the trajectory of physiological recovery and validate the causal pathway between the intervention and clinical outcomes [37,38,48]. Expanding the inclusion criteria to patients with moderate–severe inhalation injury would help define the broader applicability of this protocol [27,36]. Furthermore, integrating biomarker analysis will better elucidate the molecular mechanisms by which heparin and NAC alter the airway microenvironment. Finally, prospective studies should include structured adjudication of causes of death, and health–economic evaluations are warranted to assess the cost-effectiveness of this resource-intensive protocol against the immense costs associated with prolonged critical care for acute respiratory failure in inhalation injury.

## 5. Conclusions

A proactive, multimodal airway management protocol integrating scheduled therapeutic bronchoscopy with nebulized heparin and N-acetylcysteine is associated with a significant reduction in unadjusted 28-day mortality in burn patients with inhalation injury. The clinical efficacy of this strategy is fundamentally underpinned by a robust restoration of respiratory mechanics, characterized by a significant reduction in driving pressure and a significant increase in static compliance, which collectively indicate the successful reversal of cast-induced airway obstruction and the mitigation of ventilator-induced lung injury. Given its substantial survival benefit and favorable safety profile, this synergistic mechanical and pharmacological paradigm holds promise as a valuable therapeutic option in specialized burn units, although further validation via multicenter trials with integrated physiological monitoring remains essential to define its broader clinical utility.

## Figures and Tables

**Table 1:** Baseline characteristics of the study population (*n* = 76).

Characteristic	Control (*n* = 38)	Intervention (*n* = 38)
Age (years), median (IQR)	37.0 (31.0–48.0)	37.5 (28.0–44.0)
Male sex, *n* (%)	30 (78.9)	29 (76.3)
Height (cm), median (IQR)	168.5 (164.0–170.0)	165.0 (160.0–170.0)
Actual body weight (kg), median (IQR)	63.5 (58.0–68.0)	62.0 (54.0–67.0)
Predicted body weight (kg), median (IQR)	64.7 (60.6–66.0)	61.5 (52.4–66.0)
TBSA (%), median (IQR)	70.0 (50.0–80.0)	63.5 (50.0–87.0)
Deep burn area (%), median (IQR)	39.5 (17.0–50.0)	40.0 (17.0–55.0)
Admission PaO_2_/FiO_2_, median (IQR)	192.0 (144.0–252.0)	210.0 (173.0–280.0)
Admission carboxyhemoglobin (%), median (IQR)	1.0 (0.0–11.0)	2.0 (0.0–14.0)
Inhalation Injury Severity Grade, *n* (%)		
Grade 1 (Mild)	7 (18.4)	12 (31.6)
Grade 2 (Moderate)	13 (34.2)	10 (26.3)
Grade ≥ 3 (Severe to Massive)	18 (47.4)	16 (42.1)

**Table 2:** Fluid Resuscitation Parameters During the First 7 Days.

Parameter	Control	Intervention	*p*-Value
First 24 h total (Day 1)	14,771 (10,286–18,857) [*n* = 38]	12,000 (8726–16,800) [*n* = 38]	0.282
Total fluid volume, Day 2	6750 (4500–9000) [*n* = 38]	6000 (4500–7001) [*n* = 38]	0.109
Total fluid volume, Day 3	4996 (4001–6000) [*n* = 38]	5140 (4000–6499) [*n* = 38]	0.688
Total fluid volume, Day 4	4550 (3500–5500) [*n* = 38]	4996 (3500–6000) [*n* = 38]	0.407
Total fluid volume, Day 5	4001 (3500–4999) [*n* = 38]	4500 (3600–5000) [*n* = 38]	0.685
Total fluid volume, Day 6	4001 (3499–4999) [*n* = 32]	3750 (3150–4500) [*n* = 35]	0.203
Total fluid volume, Day 7	4000 (3499–4500) [*n* = 30]	3500 (3000–4992) [*n* = 34]	0.485

Values are expressed as median (interquartile range) in mL/24 h. For days 6 and 7, the number of patients (*n*) decreased due to ICU discharge or death. Day 1 total volume was calculated by converting the initial 24 h average hourly infusion rate into a 24 h total to ensure uniform unit presentation across the study period.

**Table 3:** Mixed-effects model estimates for respiratory mechanics and LIS.

Outcome	Effect	Coefficient (95% CI)	*p*-Value
Driving Pressure (cmH_2_O)	Intervention vs. Control	0.72 (−0.45 to 1.88)	0.227
	Time (per day)	−0.04 (−0.20 to 0.11)	0.598
	Intervention × Time	−0.56 (−0.78 to −0.34)	<0.001
Static Compliance (mL/cmH_2_O)	Intervention vs. Control	−4.30 (−7.84 to −0.76)	0.017
	Time (per day)	0.66 (0.26 to 1.06)	0.001
	Intervention × Time	2.40 (1.83 to 2.97)	<0.001
Lung Injury Score (LIS)	Intervention vs. Control	−0.10 (−0.40 to 0.20)	0.522
	Time (per day)	0.06 (0.01 to 0.10)	0.012
	Intervention × Time	−0.05 (−0.11 to 0.01)	0.091

**Table 4:** Clinical outcomes and complications.

Outcome	Control (*n* = 38)	Intervention (*n* = 38)	*p*-Value
28-day mortality, *n* (%)	31 (81.6)	22 (57.9)	0.025
VAP, *n* (%)	26 (68.4)	19 (50.0)	0.102
Septic shock, *n* (%)	25 (65.8)	23 (60.5)	0.634
Duration of MV (days), median (IQR)	12 (8–17)	15 (10–36)	0.098
ARDS, *n* (%)	18 (47.4)	12 (31.6)	0.151
Acute Kidney Injury (AKI), *n* (%)	14 (36.8)	9 (23.7)	0.201
Ventilator days in 28-day survivors, median (IQR) [*n*]	50 (16–63) [*n* = 7]	41 (34–88) [*n* = 16]	0.688
Ventilator-free days alive at day 28, median (IQR)	0 (0–5)	0 (0–11)	0.145

**Table 5:** Systemic coagulation parameters at Day 7.

Parameter, Median (IQR)	Control (*n* = 38)	Intervention (*n* = 38)	*p*-Value
PT (seconds)	13.75 (12.1–19.3)	13.35 (11.8–17.2)	0.234
aPTT (seconds)	35.9 (32.3–38.5)	34.6 (31.1–37.6)	0.301
INR	1.21 (1.1–1.39)	1.125 (1.09–1.24)	0.088
Fibrinogen (g/L)	9.09 (6.31–11.14)	8.315 (6.3–10.36)	0.467

**Table 6:** Parsimonious multivariable Cox regression analysis for 28-day mortality.

Variable	Adjusted Hazard Ratio	95% CI	*p*-Value
Intervention (vs. Control)	0.66	0.36–1.23	0.189
Age (per year)	1.04	1.01–1.07	0.005
Sex (Male vs. Female)	1.23	0.61–2.46	0.566
TBSA (per %)	1.03	1.00–1.05	0.030
Deep burn area (per %)	1.00	0.98–1.02	0.981
Inhalation injury grade (per grade)	1.61	1.03–2.53	0.037

## Data Availability

The raw data supporting the conclusions of this article will be made available by the authors on request.

## Supplementary Materials

The following supporting information can be downloaded at: https://www.mdpi.com/article/10.3390/ebj7020022/s1, File S1: CONSORT 2025 Checklist of Information to Include when Reporting a Randomised Trial.
